# Conscious While Being Considered in an Unresponsive Wakefulness Syndrome for 20 Years

**DOI:** 10.3389/fneur.2018.00671

**Published:** 2018-08-28

**Authors:** Audrey Vanhaudenhuyse, Vanessa Charland-Verville, Aurore Thibaut, Camille Chatelle, Jean-Flory L. Tshibanda, Audrey Maudoux, Marie-Elisabeth Faymonville, Steven Laureys, Olivia Gosseries

**Affiliations:** ^1^Department of Algology and Palliative Care, University Hospital of Liege, Liege, Belgium; ^2^GIGA-Consciousness, Sensation & Perception Research Group, University of Liege, Liege, Belgium; ^3^GIGA-Consciousness, Coma Science Group & Neurology Department, University Hospital of Liege, Liege, Belgium; ^4^Neuromodulation Center, Spaulding Rehabilitation Hospital, Harvard Medical School, Boston, MA, United States; ^5^Laboratory for NeuroImaging of Coma and Consciousness-Department of Neurology, Massachusetts General Hospital, Harvard Medical School, Boston, MA, United States; ^6^Department of Radiology, University Hospital of Liege and University of Liege, Liege, Belgium; ^7^Otorhinolaryngology Head and Neck Surgery Department, University Hospital of Liege, Liege, Belgium

**Keywords:** disorders of consciousness, misdiagnosis, locked-in syndrome, unresponsive wakefulness syndrome, MRI, PET, EEG, vegetative state

## Abstract

Despite recent advances in our understanding of consciousness disorders, accurate diagnosis of severely brain-damaged patients is still a major clinical challenge. We here present the case of a patient who was considered in an unresponsive wakefulness syndrome/vegetative state for 20 years. Repeated standardized behavioral examinations combined to neuroimaging assessments allowed us to show that this patient was in fact fully conscious and was able to functionally communicate. We thus revised the diagnosis into an incomplete locked-in syndrome, notably because the main brain lesion was located in the brainstem. Clinical examinations of severe brain injured patients suffering from serious motor impairment should systematically include repeated standardized behavioral assessments and, when possible, neuroimaging evaluations encompassing magnetic resonance imaging and ^18^F-fluorodeoxyglucose positron emission tomography.

## Introduction

We here present the case of a 41-year-old man who was considered in an unresponsive wakefulness syndrome (UWS; previously referred to as “vegetative state”) for 20 years. In this section, we first review his medical history then we report the clinical and neuroimaging evaluations that were performed in our center 20 years after his brain injury.

### Patient's history

In 1992, the patient sustained a severe traumatic brain injury as a result of a car accident. He had no previous significant medical history. On admission to a general hospital, the Glasgow Coma Scale (1) total score was 4/15 and both pupils were in myosis. Babinski reflex was present bilaterally. The patient was intubated and mechanically ventilated. Brain CT scan revealed left parietal, basal ganglia, and retro-pontic hemorrhages. The EEG displayed a non-reactive global slowing of basic rhythms without paroxystic activity. The patient was tracheotomized, received nasogastric feeding and left the intensive care unit 24 days later with the diagnosis of “coma vigil.” Six weeks after the insult, the treating nurse of the neuropsychiatry department reported that the patient had moved his right hand to command, but this observation did not change the clinical diagnosis and it was never reported on later occasions. Two epileptic seizures were observed 6 months post-injury. The tracheal tube was removed 8 months after the brain trauma. Neurological examination performed 9 months post-onset reported spontaneous eye opening without reproducible response to command, and concluded to a state of “irreversible coma vigil” (i.e., permanent vegetative state). One year and 5 months post-injury, he was transferred to a chronic nursing care home with the diagnosis of “comatose state.” The patient did not receive physiotherapy, speech therapy or occupational therapy. No stimulation or rehabilitation treatment was reported by the medical team in the nursing home.

Twenty years after his brain injury, the patient was transferred to our neurology department for a diagnostic evaluation as requested by the general practitioner of his nursing care home. The request was initiated by the family of the patient who was staying in the same room who had the impression that he was conscious. The diagnosis on referral was “coma vigil.” Pharmacological treatment included diphantoine (4 × 100 mg/d—antiepileptic), mirtazapine (1 × 30 mg—antidepressant) and lormetazepam (1 × 2 mg/d—sedative benzodiazepine). Medication was not modified during the week of assessment. Hetero-anamnesis was limited given that no family could be reached.

### Clinical assessments

The patient's consciousness level was assessed with the Coma Recovery Scale-Revised [CRS-R, (2)]. This scale is currently considered the most validated and sensitive method for identifying behavioral signs of awareness and thus better diagnose between UWS, minimally conscious state and emergence of the minimally conscious state (2–5). It consists of six subscales: auditory, visual, motor, oromotor and verbal functions, communication, and the level of arousal. The 23 items are ordered by degree of complexity, ranging from reflexive to cognitively mediated behaviors. We recently reported that a minimum of five CRS-R assessments conducted within a short time interval (e.g., 2 weeks) was necessary to reduce misdiagnosis (6). Here, the patient underwent seven CRS-R assessments in a period of 1 week; these were performed by a team of experimented examiners at different moments of the day, and in similar environmental conditions. To assess the patient's spatio-temporal orientation, we asked on one occasion some questions of the Mini Mental State Examination [MMSE, (7)].

Pain perception was also assessed once with the Nociception Coma Scale-Revised [NCS-R, (8)], which consists of three subscales evaluating motor, verbal, and facial expression responses; each subscore ranges from 0 to 3 (maximum total score of 9). Additional physiotherapy and otorhinolaryngology examinations were performed during the week of hospitalization.

Spontaneously, the patient showed eyes opening, chewing, left wrist and leg movements as well as visual fixation and visual pursuit; these two latter are considered as signs of consciousness (9, 10). The CRS-R examinations straightforwardly showed that the patient was not in a UWS (**Table 2**). The CRS-R total score varied between 12 and 17. During every single assessment, the patient was able to repeatedly follow simple commands (e.g., close your eyes, open your mouth, lift your thumb). On two consecutive assessments, he could also functionally communicate (i.e., being able to systematically and accurately answer simple questions using a “YES/NO” codes), which means that he emerged from the minimally conscious state. The first time, the patient correctly answered the CRS-R visual questions using YES and NO cards. The second time, he responded accurately to self-related questions using a buzzer (i.e., buzz once to say yes). On three other assessments, the patient presented an intentional non-functional communication [i.e., clearly discernible communicative responses occurred on at least two out of the six questions, irrespectively of accuracy; (2)]. During all these assessments, we tried different codes of communication with the patient, such as point out YES/NO cards or rise your thumb to say YES/do not move your thumb to say NO, to finally observe that the best way to communicate was with visual fixation of YES/NO cards on the vertical axis.

Furthermore, the patient showed visual pursuits (on vertical and horizontal planes on all assessments), automatic motor responses (e.g., touch his mouth), anticipation and grimaces after nociceptive stimulations, and objects localization.

When assessing his spatio-temporal orientation using YES/NO cards, the patient was able to correctly indicate his first and last name, the names of his roommate and the mother's roommate. He was, however, not able to give his age, to locate the hospital, neither the exact date (day, month, year) nor the season.

NCS-R assessment highlighted withdrawal flexion, groaning and grimacing in response to nociceptive stimulation (total score of 5), as well as abnormal stereotyped posture and oral movements during nursing cares (total score of 2). Physiotherapy assessment showed spasticity in flexion in the right superior limb and abnormal extension with internal rotation in the left superior limb. The feet were fixed in equine varus positions and the knees flexions were limited. The head suffered from a vicious position in deviation to the left. Otorhinolaryngology examination evidenced significant spasticity of the entire cephalic segment, major spasticity of the whole neck muscles with the impossibility to reduce left deviation. A left saliva drooling was observed but the velar reflex and nausea reflex were absent. A naso-pharyngo-laryngeal fibroscopy showed that the nasal cavities, the pharynx and the larynx were structurally normal. The laryngeal sensitivity was reduced and no cough reflex could be evoked. Food testing was attempted but was impossible to perform due to a deficient oral phase.

### Neuroimaging assessments

For structural MRI, a high-resolution T2-weighted image was acquired (25 slices; repetition time = 3,000 ms, echo time = 88 ms, voxel size = 0.9 × 0.9 × 3 mm^3^, field of view = 220 × 220 mm^2^) on a 3 Tesla scanner (Siemens Trio, Siemens Medical Solutions, Erlangen, Germany). Diffusion tensor images (DTI) were acquired using an EPI sequence (TR = 5,700 ms, TE = 87 ms, 45 slices; slice thickness = 3 mm, gap = 0.3 mm, matrix size = 128^*^128) and sensitized in 64 non-collinear directions using a *b*-value = 1,000 s/mm^2^ and two *b* = 0 images. Data were acquired and analyzed similarly to our previous studies (23, 24). Images were processed using the FMRIB Software Library (FSL; version 4.1.2; http://www.fmrib.ox.ac.uk/fsl). Fractional anisotropy and mean diffusivity maps were obtained using FSL diffusion toolbox (25).

**Figure 1:**
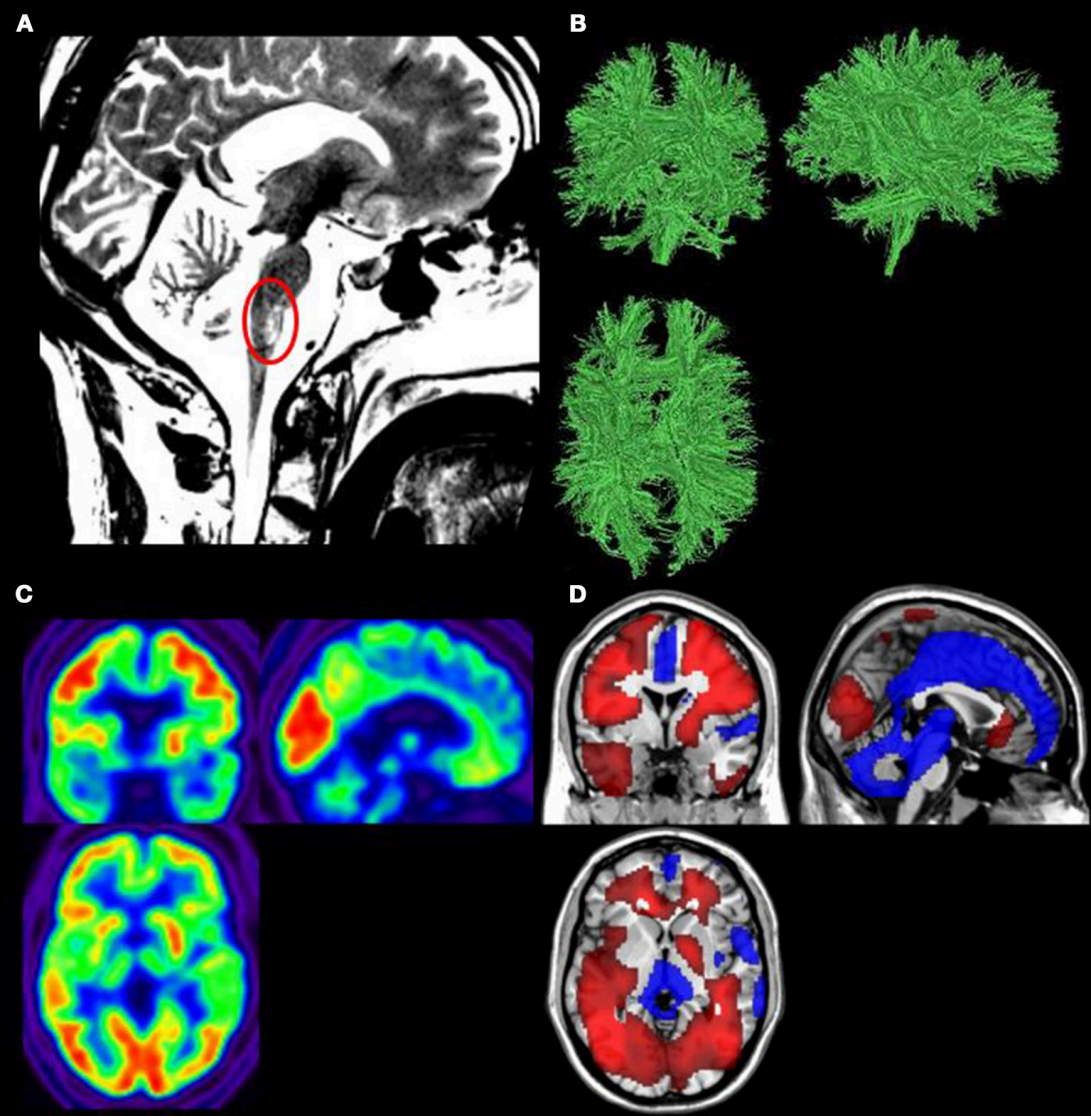
**(A)** Structural magnetic resonance imaging (MRI) showed the mesencephalic tegmentum lesion (red circle). **(B)** Diffusion tensor imaging (DTI) showed white matter structure preservation. **(C)** ^18^F-fluorodeoxyglucose positron emission tomography (FDG-PET) demonstrated a global cerebral metabolism preservation. **(D)** Areas in which FDG–PET finds significantly impaired (blue) or preserved (red) metabolism compared to controls (*p* < 0.05, uncorrected).

Structural MRI showed post-traumatic diffuse axonopathy lesions in the right middle cerebellum peduncule, right cerebral peduncule, left lenticular nucleus, corpus callosum, right superior frontal gyrus, and mesencephalic tegmentum (Figure 1A). There was no parenchymatic atrophy.

DTI showed a relative preservation of the white matter structure (Figure 1B). The global fractional anisotropy was estimated at 0.32 (normal range in healthy control subjects between 0.35 and 0.59).

For resting cerebral ^18^F-fluorodeoxyglucose positron emission tomography (FDG-PET), data were also acquired and analyzed as in our previous studies (19, 26). Before and after injection of 300 MBq of FDG, the patient was kept awake in the dark for 30 min and was then scanned on a Gemini TF PET-CT scanner (Philips Medical Systems). Data was preprocessed using spatial normalization, smoothing (Gaussian kernel of 14 mm full width at a half maximum) and proportional scaling, implemented in Statistical Parametric Mapping toolbox, SPM 12 (www.fil.ion.ucl.ac.uk/spm). The design matrix modeled the patient and 34 age-matched healthy controls' PET-scans. We used a significance threshold of *p* < 0.05 uncorrected in all contrast for single subject analyses.

Results showed a preservation of 99.6% of the patient's global brain metabolism as compared to healthy subjects (Figure 1C). Preserved brain regions encompassed the whole fronto-temporo-parietal cortex bilaterally. Hypometabolism was observed in the mesiofrontal region, the thalamus bilaterally, the brainstem and the cerebellum (Figure 1D).

Both MRI and PET data show a brainstem lesion, which is observed in patients with locked-in syndrome [(27), Figures 1A,D].

A clinical EEG was also performed using 19 electrodes and interpreted by a certified neurologist. Results showed bilateral alpha activity and 8–10 Hz activities on all derivations without any paroxysmal activity.

The days of neuroimaging assessments (as well as the NCS and the MMSE) are reported in Table 1.

**Table 1:** Behavioral responses of the patient assessed with the Coma Recovery Scale-Revised.

**Day of assessment**	**1**	**2**	**3**	**4**	**5**	**6**	**7**
**Other evaluations performed the same day**		**EEG**	**PET NCS**	**MMSE**	**MRI**		
**AUDITORY FUNCTION**							
4—Consistent Movement to Command^*^ (4/4 responses of 2 different commands)				x		x	x
3—Reproducible Movement to Command^*^ (3/4 responses of 1 command)	x	x	x		x		
2—Localization to Sound	x	x	x	x	x	x	x
1—Auditory Startle	x			x			
0—None							
**VISUAL FUNCTION**							
5—Object Recognition^*^		x	x	x	x	x	x
4—Object Localization: Reaching^*^			x	x			
3—Visual Pursuit^*^	x	x	x	x	x	x	x
2—Fixation^*^	x	x	x	x	x	x	x
1—Visual Startle	x	x	x	x	x	x	x
0—None							
**MOTOR FUNCTION**							
6—Functional Object Use^#^							
5—Automatic Motor Response^*^					x		
4—Object Manipulation^*^							
3—Localization to Noxious Stimulation^*^							
2—Flexion Withdrawal	x			x	x	x	x
1—Abnormal Posturing		x	x	x			
0—None/Flaccid							
**OROMOTOR/VERBAL FUNCTION**							
3—Intelligible Verbalization^*^							
2—Vocalization/Oral Movement	x	x	x	x		x	x
1—Oral Reflexive Movement	x	x	x	x		x	x
0—None					x		
**COMMUNICATION**							
2—Functional: Accurate^#^				x	x		
1—Non-Functional: Intentional^*^		x				x	x
0—None	x		x				
**AROUSAL**							
3—Attention							
2—Eye Opening w/o Stimulation	x	x	x		x	x	x
1—Eye Opening with Stimulation				x			
0—Unarousable							
Total score	12^*^	14^*^	13^*^	16^#^	17^#^	16^*^	16^*^

* *Denotes MCS*.

# *Denotes emergence of MCS*.

## Background

Despite recent advances in our understanding of disorders of consciousness and the redefinition of nosological distinctions between altered states of consciousness, diagnosis of severely brain-damaged patients continues to represent a major clinical challenge. If neuroimaging techniques support clinical examinations and help to improve the accuracy of the diagnosis of altered state of consciousness, behavioral assessment remains the principal method used to detect awareness in these patients (28). Nowadays, standardized scales such as the CRS-R (2) are validated to assess the level of consciousness of these patients. In addition, series of studies have reported that specific clinical tools [e.g., using a mirror to assess visual pursuit (9, 10) or the own name to assess localization to sounds (29)] can increase the chance of observing behavioral responses. In spite of these developments, clinical practice shows that disentangling reflexive from voluntary behaviors can still be very difficult.

Several misdiagnosis studies have been described in patients at an early stage after severe brain damage, as well as in the long-term care (Table 2). Some studies reported cases of patients considered unconscious while they actually presented behavioral signs of consciousness when assessed more thoroughly (5, 6, 11–13, 15, 16, 19–22). Other studies recount cases of patients who were considered unconscious at the bedside but who were actually found to be conscious with neuroimaging techniques, and some of these patients could even communicate with adapted communication code (18, 19, 30–32). Different factors can explain the high rate of diagnosis errors in patients with disorders of consciousness: the lack of knowledge about the diagnosis criteria and terminology, the absence or misuse of standardized assessment scale, the use of insensitive tools, the patients' perceptual and/or motor deficits, the presence of language impairment, the fluctuating arousal level, and the presence of pain or sedative drugs (33, 34).

**Table 2:** Studies reporting misdiagnosis of UWS.

**References**	**Method**	**Number of patients**	**Number of patients misdiagnosed**	**% of misdiagnosis**	**Etiology**	**Initial diagnosis**	**Correct diagnosis**	**Duration of the misdiagnosis**
Tresch et al. 11	Clinical consensus vs. Author's examination	62	11	18%	NA	UWS	MCS	Chronic (>1 year)
Childs et al. 12	Clinical consensus vs. Author's examination	49	18	37%	14 TBI 4 NTBI	UWS	MCS	1–3 days
Andrews et al. 13	Clinical consensus vs. RLA 14	40	17	42%	10 TBI 7 NTBI	UWS	15 MCS 2 EMCS	Range 2–175 days
Tavalaro and Tayson 15	Clinical consensus vs. Family and nurses impression	1	1	NA	Stroke	UWS	LIS	6 years
Gill-Thwaites and Munday 16	Clinical consensus vs. SMART 17	60	27	45%	21 TBI 39 NTBI	UWS	“Higher level of functioning than VS”	Within 4 months
Schnakers et al. 5	Clinical consensus vs. CRS-R	44	18	41%	39 TBI 64 NTBI	UWS	MCS	NA
Lukowicz et al. 18	Clinical consensus vs. Family impression	1	1	NA	Brain tumor	“Unconscious terminal stage”	LIS	16 years
Stender et al. 19	Clinical consensus vs. CRS-R	51	18	35%	TBI and NTBI	UWS	MCS	Mean duration of UWS: 2 years and 3 months
Sattin et al. 20	Experience rater CRS-R vs. CRS-R with person responsible of patients	92	15	16%	25 TBI 67 NTBI	UWS	MCS	Mean duration of UWS: 2 years and 6 months
van Erp et al. 21	Clinical consensus vs. CRS-R	41	17	41%	TBI and NTBI	UWS	MCS	Mean duration of UWS: 5 years
Cortese et al. 22	Morning CRS-R vs. Afternoon CRS-R	7	2	30%	2 TBI 5 NTBI	UWS	MCS	1.8–6.2 years
Wannez et al. 6	1 CRS-R vs. 5 CRS-R	62	22	35%	TBI and NTBI	UWS	MCS	Mean time since injury 4 years

*UWS, unresponsive wakefulness syndrome (vegetative state—VS); MCS, minimally conscious state; EMCS, emergence of minimally conscious state; LIS, locked-in syndrome; TBI, traumatic brain injury; NTBI, non-traumatic brain injury; NA, non applicable*.

Studies have highlighted the importance to properly diagnose clinical entities because patients in minimally conscious state retain some preserved capacities for cognitive processing, which is not the case in patients with UWS who only show reflex behaviors (35–37). In addition, outcome and responses to treatment of minimally conscious patients seem more favorable than those in a UWS (38–40). Clinical decisions about pain management and end-of-life are also influenced by the diagnosis (41–43). A similar yet very different group of patients are those with a locked-in syndrome [LIS; (27)]. Patients with LIS are completely conscious but they have no muscle control due to a disruption of the brainstem's cortico-spinal pathways. However, most of these patients recover minimal motor function with time, and some may even recover almost fully, as it is sometimes the case with *incomplete LIS* (44, 45). On the other hand, some patients with LIS have other brain lesions outside the brainstem which might induce cognitive impairments (46, 47).

## Discussion

Our standardized-repeated behavioral assessments detected signs of consciousness and functional communication at the patient's bedside, which indicates that the patient emerged from the minimally conscious state. The neuroimaging results confirmed that the patient was conscious and that he actually was in a LIS due to a lesion in the brainstem. Because the patient could move more than a classical LIS, the diagnosis of incomplete LIS was finally made.

This patient had a brain injury 20 years before his admission to our center and he was misdiagnosed as being unconscious all these years when he was in fact fully conscious. The lack of knowledge about differential diagnosis of disorders of consciousness during this time period can explain that the patient received the diagnosis of “coma vigil” or “vegetative state.” The LIS was defined in 1966 (48), while criteria of the minimally conscious state and emergence of this state were defined much later, in 2002 (49). Moreover, 20 years ago, behavioral assessment of consciousness were limited to very few scales such as the Glasgow Coma Scale, which is not sensitive enough to detect small signs of consciousness (4). Our clinical practice shows that once stamped with the diagnosis of UWS, it is often difficult to change the label, and the first signs of recovery of consciousness can be missed. The negative associations intrinsic to the term “vegetative state” can result to diagnostic errors and can also lead to potential effect on the treatment and care (37).

This case report also shows how difficult it can be to properly assess signs of consciousness and evaluate cognitive impairment in severely brain-injured patients suffering from profound physical disabilities. In order to detect consciousness in these patients, we are limited to make inferences about the presence or absence of motor responses. Behavioral examination is very challenging because observed movements may be small, inconsistent and easily exhausted, potentially leading to diagnostic errors.

On one hand, the American Congress of Rehabilitation Medicine (50) defines the following neurobehavioral criteria of the LIS: eyes opening, evidence of basic cognitive abilities, quadriparesis or quadriplegia, as well as eyes movements way of communication, usually escort by lesions of the ventral pons. In addition, intact intellectual abilities characterize the classical LIS (46). On the other hand, emergence from the minimally conscious state is defined by the demonstration of either functional communication or functional use of objects, on two consecutive assessments. Our patient showed spontaneous eyes opening and severe motor impairment that could be related to quadriparesis. Communication, which was detected and could be possible via eye movements, was not easily reproducible: out of seven assessments, the patient was able to functionally communicate only on two consecutive assessments while a non-functional intentional communication was detected on three evaluations. Even if the patient presented an eye-movement-based communication, the diagnosis of incomplete LIS is challenging at the behavioral level because his communication responses fluctuated a lot. In addition, we should consider that the patient's deficit in spatio-temporal orientation (such as his inability to report the exact date or to locate the hospital) could be related to his 20-years-long impossibility to read a calendar or to be informed about the world outside his room rather than to a cognitive impairment. Inconsistency of behavioral responses and difficulties to correctly answer to orientation questions could also be the result of a lack of stimulation for the past 20 years.

At the neuroimaging level, structural MRI, DTI, and FDG-PET results highlighted a preservation of global cerebral metabolism and cerebral white matter combined with a lesion in the brainstem. The brain lesions observed with the neuroimaging tools, specifically in the brainstem, are typically observed in patients with LIS (51), with additional brain lesions (46).

In 33% of cases, a previous study showed that it was the relatives of the patient with LIS who were the first to detect consciousness and ability to communicate (52). In addition, guidelines emphasize the importance that the diagnosis should be made by involving information from family members or other persons who see the patient regularly (53). Other studies have also insisted on the critical role of the family or of a close relative in the assessment of patients (54).

The story of the patient we reported here is marked by an important social isolation. Indeed, since his accident, his family and friends were disengaged from the care and his general condition. The only people in daily contact with him were members of the medical staff. Since 1994, the patient was in a long care nursing home. Even if nurses knew him very well after all these years, they always referred to him as a “vegetative state.” The intrinsic negative connotation of the term “vegetative state” can lead to situations where the patients' relatives interpret this diagnosis as he is no longer a human being (but more a “vegetable”), and that there is no hope of recovery (55). The “unresponsive wakefulness syndrome” terminology was thus adopted to be more descriptive of the actual state of these patients and preventing the use of a pejorative term (35). In addition, even if the medical team usually strive to maintain these people's rights as human beings and treat them with respect, it is difficult to be optimistic and adopt a positive attitude during years when patients are very low responsive.

Recent advances in technologies have demonstrated the possibility of establishing binary communication with severe brain injury patients using solely mental processes. These brain computer interfaces (BCI) technologies have employed neural responses detectable with EEG, to provide patients with motor impairments the ability to control a computer. These interfaces usually drive software for simple communication, or control devices that influence some aspect of the patient's external environment. In addition, they provide the patient with valuable real-time feedback on their performance, enabling them to learn how to use the interface better over time [for a review, see (56, 57)]. Recently, a novel BCI based on steady-state visually evoked potential or functional near-infrared spectroscopy were developed, tested and validated with patients in LIS (58, 59). These BCI technologies could benefit to patients who are severely motor impaired and potentially allow clinicians to detect signs of consciousness and elaborate communication with these very challenging patients.

One can point as limitation that neuropsychological testing is lacking in the evaluation of this patient. Neuropsychological testing and specifically the ones adapted for non-communicative patients (46, 60) would have been useful to better determine the patient's cognitive abilities. Another limitation is the lack of assessment during these 20 years. Indeed, the patient may have recovered slowly over these years with no expert to assess his progress. One can also argue that the patient was at some point in a functional locked-in syndrome [i.e., patients with a dissociation between extreme motor dysfunction and preserved higher cortical functions identified only by functional imaging techniques; (36)] but misdiagnosed as being in UWS because neuroimaging techniques were not available at that time to detect consciousness (61).

## Concluding remarks

In conclusion, this report emphasizes both the complex nature of patients with severe brain injury and the necessity to use validated sensitive techniques to make an accurate diagnosis. Accurate diagnosis in the early stages will determine cares and patients management after their brain injury. If misdiagnosis of UWS is frequent for patients who actually are in a minimally conscious state, this misdiagnosis is, even if less frequent, still observed in patients who are in fact totally conscious like LIS patients. Since behavioral assessments remain the gold standard to detect consciousness, clinicians should be cautious in the scales they use to assess patients, as well as to additional cognitive impairments as a consequence of specific brain lesions. To date, the most sensitive and validated scale is the CRS-R (2). The number of CRS-R assessments has an impact on the clinical diagnosis of patients since a lack of repeated examinations in patients with DOC can lead to an underestimation of patients' level of consciousness (6). It was recently demonstrated that a minimum of five CRS-R assessments is required for a reliable clinical diagnosis in DOC (6).

This case report also emphasizes the need for neuroimaging in the assessment of consciousness to confirm or refute the clinical diagnosis. In addition, we should notice that the diagnosis of UWS of this patient was maintained because he was abandoned early in a chronic setting, where there was no adequate expertise in the assessment of persons with disorders of consciousness and in a condition of social isolation. A close collaboration and involvement of family should be systematic in cares and assessments of patients with disorders of consciousness.

## Ethics statement

The study was approved by the Ethics Committee of the Faculty of Medicine of the University of Liège, Belgium.

## Author contributions

AV and OG were responsible for acquisition, analysis and interpretation of data and drafting the article. VC-V was responsible for interpretation of data and drafting the article. AT, CC, J-FT, and AM were responsible for acquisition and interpretation of data and revising the article. M-EF and SL were responsible for interpretation of data and revising the article.

### Conflict of interest statement

The authors declare that the research was conducted in the absence of any commercial or financial relationships that could be construed as a potential conflict of interest.
